# Better, and ‘healthier’ decision making through information technology: conference report from the Health 2.0 conference in San Francisco, September 2011

**DOI:** 10.3332/ecancer.2011.242

**Published:** 2011-11-24

**Authors:** S Camporesi

**Affiliations:** 1 Centre for the Humanities and Health, Strand Campus, King’s College London, WC2R 2LS, UK; 2 Current address: Department of Anthropology, History and Social Medicine, 333 California Street, Suit 475, San Francisco CA 94143-0850

## Abstract

San Francisco served as the host city for the 5th Annual Health 2.0 conference on 25–27 September 2011, which gathered more than 1,500 people interested and actively involved in innovation in health care through information technology. The author attended this conference and in this paper is presenting key insights, concepts, and news from the cutting edge of Health 2.0: from new tools to redesign medical data; to synergistic interactions between web 2.0 tools and public health; to the use of the internet as a positive catalyst for behavioral change to improve health and lifestyle; to direct to consumer genetic testing that drive a new, community-driven approach to research and to electronic health records adoption and data liberation. The main thread linking all of these initiatives is a participatory approach to health that involves all relevant stakeholders: from patients and their families, to physicians and health professionals, to nurses and caregivers, to insurance and other health providers, working together toward the common aim of a better health care.

## Introduction: what is ‘Health 2.0’?

San Francisco served as the host city for the 5th Annual Health 2.0 conference on September 25–27 of this year, which gathered more than 1,500 people interested and actively involved in innovation in health care through information technology [1]. The author attended this conference, and in the following sections is sharing some key insights, concepts, and news from the cutting edge of Health 2.0—a global movement born in the Bay Area but that is rapidly changing the face of health care as we know it today.

What exactly is Health 2.0? For **Indu Subaya** (cofounder and CEO of Health 2.0, http://indusubaiya.com), three features are essential for defining Health 2.0: (a) a easily delivered and cloud-centered technology, (b) heavy focus on user experience through design and usability, and (c) use of data from multiple sources that improve private and public health through lowering cost and better decision making. **Matthew Holt** (cochair and cofounder of Health 2.0, http://www.matthewholt.net/) added to Indu that one of the missions of Health 2.0 is *data liberation*, i.e. the patients should be able to access freely their own data. Data liberation, to be achieved through patient access to their electronic health records (EHRs), was indeed one of main themes of this year’s conference.

**Figure 1:**
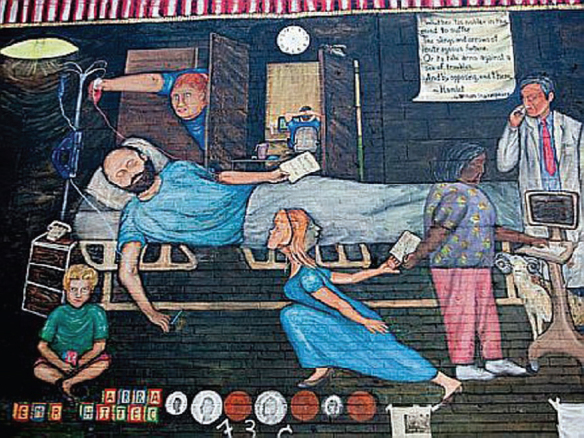
‘73 cents’ by Regina Holliday. Reproduced with kind permission from the artist.

Data liberation is also the self-proclaimed mission of **Regina Holliday**, a DC-based patient advocate and artist, whose patient advocacy mission was inspired by her husband Freddie’s struggle to find appropriate care during 11 weeks of continuous hospitalization at five different facilities when he was terminally ill with cancer. Since the death of her husband, Regina has been working as an artist to do everything she possibly can so that patients would have the right to access their own electronic medical records and therefore, have access to a better health care through different providers and hospitalizations. After Freddie’s ‘avoidable’ death—as put by Regina—in 2009, Regina began painting a mural entitled ‘73 cents’ (Figure 1), where he is positioned like Marat in the painting ‘Death of Marat’ by Jacques-Louis David (Marat, friend of Robespierre, and Jacobin deputy to the Convention, was stabbed to death in his bathtub on 13 July 1793, by a young Royalist named Charlotte Corday. This is how he was painted by Jacques-Louis David and he holds in his hand a paper that says ‘Go after them, Regina,’ referring to the system of health-care providers and insurance companies who made it so difficult for him to have proper access to health care while he was changing facilities and hospitals in search for the best possible care.

The participatory nature of Health 2.0 and the need to include all stakeholders in order to achieve the best possible treatment were highlighted by **Lygeia Ricciardi**, Senior Policy Adviser at the Office of the National Coordinator of Health IT (ONC), who defines Health 2.0 as ‘*participatory health and healthcare for patients, consumers and caregivers, as well as more traditional members of the health team, as doctors, nurses, other providers working together collaboratively to improve care*’. The http://www.healthit.gov website launched by ONC in September with the appropriate tagline, “Putting the ‘I’ in health IT” is a part of ONC’s nationwide campaign to raise awareness of the value and benefits of digital health data, and to drive patient engagement. ‘*What I am doing is making it easier for consumers and patients to get basic access to their own health information*’ says Lygeia. In other words, data liberation. As put by Lygeia, ‘*We are trying to make [data liberation] as easy as pushing a button*’.

In line with making data liberation as easy as pushing a button, the **Rainbow Button** is an initiative promoted by the satellite symposium **Patients 2.0** that takes its name from the Blue Button initiative promoted by the federal government as a means for Veterans to download their personal data. The idea then evolved at Health 2.0 meetings into adoption of the Blue Button initiative to the private sector, and inclusion of a Green Button where a user can de-identify his or her own health data, and of a White Button that would make it easy to share information and personal data with members of the medical team or with other patients.

There has been a lively debate in the United States whether electronic health records actually make a difference in terms of better health-care outcomes. In a paper recently published on NEJM, the authors calculated the percentage-point difference between EHR-based and paper-based practices with respect to achievement of composite standards for diabetes care and outcomes. Across all insurance types, race or ethnic group, age, sex, income and level of education, results showed that EHR-based practices were associated with significantly higher achievement of care and outcome standards, and greater improvement in diabetes care, therefore definitely tilting the scale of the debate in favor of adopting EHRs [2].

## Engaging Patients 2.0 for data liberation

Over the last year, the movement Patients 2.0 has brought together hundreds patients and advocates who have contributed with their narratives about using, sharing, and cocreating health data [3]. At Health 2.0 the Patients 2.0 session was chaired by **Jane Sarasohn-Kahn**, health economist and management consultant working at the intersection of health and technology and often collaborating with California HealthCare Foundation on projects that focus on health consumers and technology. The session aimed to define ways empowered patients can influence and shape future health policy and resulted in a **manifesto** consisting with the following elements:

- Engage emotionally and analytically;
- Let patients control their own data and add to it;
- Include the patient’s voice in decision making concerning care, and policy;
- Engage people and provide services where they ‘are’ (i.e. facilitate the breaking down of linguistic, cultural, and geographical barriers);
- Recognize all stakeholders: patients, caregivers, providers as equal partners with different roles;
- Network everyone, including those who are not online.

For Jane, Health 2.0 is the ‘Ability to leverage the social web for health applications, for all stakeholders in the health ecosystem: patients, consumers, caregivers, and around them all the other stakeholders: doctors, hospitals, providers, pharma…payers, employers, unions etc.’ Basically, any organization that has a role in shaping health and leverages, the social web is especially to empower patients. As an active blogger on http://healthpopuli.com, Jane is personally involved in promoting data liberation by telling people about their rights to data and health information, and by promoting health literacy. Jane is also committed to empowering people on a daily basis to make better decisions, by paying close attention to Observations of Daily Living (ODLs) that are at least as valuable as what comes out of a laboratory test. Jane is also encouraging the development of fun and engaging interfaces to get the data out.

## Redesigning and enhancing access to medical data

Which brings us to one of the big names presenting at the Health 2.0 conference, namely **Thomas Goetz**, chief editor of Wired. Thomas argued that it is time to redesign medical data to make them more accessible to patients, and was also one of the 2011 finalists of ***Launch!***, a challenge where for the first time a company demoes at a conference to introduce a product that is either coming out soon or is in a very limited beta version. Thomas Goetz, who has written extensively about making laboratory results reports more useful, presented a demo, as 1 + 1 Labs, of a product that does just that [4]. Another tool aimed at redesigning medical data is the **Drug Facts Box** [5]. Developed by Dr Lisa Schwarts and her husband Steven Woloshin at Dartmouth Institute for Health Policy and Clinical Practice in New Hampshire, it is one page summary of how the drug works. It is modeled after the Nutrition Facts label and it makes a few very clear statements: what is this drug for? Who is this for? Who should take it? And who should not? In addition, the label tells the consumers what the clinical trials showed. At the time of writing, FDA is considering a recommendation to require Drug Facts on pharmaceutical labels.

## Synergistic interactions between web 2.0 and public health

As narrated in his recently published book ‘The Decision Tree: Taking Control of Your Health in the New Era of Personalized Medicine’ [6], Thomas recognized the existence of a chasm between the impact of information technology and the goals of public health, and a great potential in combining the two, when he was working as an editor at Wired, and commuting to Berkeley every day to attend classes in a Master’s in Public Health program. Indeed, public health, aimed at prevention at the population level, would tremendously benefit by being coupled to web 2.0 tools, which Goetz defines as the ‘*new generation of internet tools that draw on the individual’s own participation and collaboration to create a highly personalized online experience*’. As put by **Leonard Syme**, Professor Emeritus of Epidemiology and Community Health/Human Development at Berkeley and quoted by his former student Goetz in his book, ‘*The whole field of public health is based on the idea that we can identify the risk factors for disease, share the data with the public, and everybody will go home and change their behavior*’. Well, it turns out it does not actually work like that for several reasons, from the fact that for complex diseases it is not trivial task to identify the risk factors, and the relevance of the factors for the disease; to the fact that it is very difficult to change engrained behaviors and habits; to the fact that medicine so far has put little effort into stopping people entering a path that leads toward disease (i.e. acting on changing what we can change, as lifestyle). All the while, most of the emphasis is traditionally placed on getting out of the path those who are already on it, which is much more difficult! Such emphasis, says Syme, must shift toward prevention and changing people’s behaviors for better and healthier decision making. How to achieve that is one of the Health 2.0 challenges.

This year, Health 2.0 is also the recipient of two major ONC contracts under the investing in Innovation (i2) program, both under the larger umbrella of the America Competes Reauthorization Act, signed into law by President Obama in January 2010. These two contracts aim at fostering innovation in health care by giving prizes and awards for creating applications that use data from different public sources. **Damien Leri** was the winner of one of the Health 2.0 challenges and is the creator of projects employing data for public health purposes. Damien received a B.A. in Psychology and an MS in Education at the University of Pennsylvania, where is currently pursuing a Master of Public Health. On his website, Damien has a quickly growing list of projects related to health information technology and public health in the United States [7], spanning from an interactive tool for exploring data related to obesity in the United States; to an interactive comparison of water and sanitation globally, aptly called ‘Deadly Showers,’ linked with certain health outcomes across countries; to a multilingual web application called ‘Prepped Kids’ for hyper-local mapping of preschools, pediatric and family medicine, and supporting resources. All these projects and others are collected on ‘Mapping Health’, a website that creates original data visualizations and apps for exploring or managing public health data and that reaches an audience ranging from academics and health professionals to patients and health-care consumers [8].

## ‘Healtier’ decision making through web 2.0 tools

Better, and ‘healthier’ decision making through web 2.0 tools was indeed another of the take-home messages of the 3-day conference. One of the very first examples of decision-tree-based tools that can help us make better decision in health, developed by Peter Ravdin then at the University of Texas at San Antonio, was ‘***Adjuvant!***’ [9], a tool designed to help physicians dealing with treatment decisions for women with breast cancer. How does *Adjuvant!* work? A physician logs on and fills in data about the patient’s pathology report (e.g., patient age, tumor size, nodal involvement, histologic grade, etc), then the software’s output is a series of charts that lay out the risks and benefits of various therapies following surgery. The purpose of *Adjuvant!* is to help health professionals make estimates of the risk of negative outcomes (cancer-related mortality or relapse) without systemic adjuvant therapy (i.e. usually chemotherapy, hormone therapy, or both), estimates of the reduction of these risks afforded by therapy, and risks of side effects of the therapy. Though initially focusing on breast cancer, the *Adjuvant!* online website now includes models for colon and lung cancer. All of us, as patients, can make better choices for our health based on the inputs we have, which can be codified in web 2.0 tools. In this regard, ‘Health’ can be seen as a decision-making phenomenon, and while a decision tree is not a panacea to our health problems, it can help us to recognize what, in fact, we do have control over versus what we do not have control over. Given our very human desire for control in the important decisions of our life (and health decisions definitely fall in this realm!), a decision tree can become a very valuable tool to diminish the uncertainty inherent in our health-related decisions. As put by Goetz, a decision tree ‘*puts science within reach, it reorients it to personal circumstances, and it turns data into actionable information.*’ By acknowledging and taking into account the inherent uncertainty of our decisions, a decision tree can help us get the best possible health outcomes given the inputs we have. If the goal of perfect health is impossible to reach, then it helps us readjust and set new goals.

Recent years have seen a plethora of prediction models of which *Adjuvant!* was only one of the first examples. As argued by Andrew J. Vickers, Department of Epidemiology and Biostatistics at Memorial Sloan-Kettering Cancer Center, New York, [10] though there can be no doubt that prediction models are here to stay and be used as a decision-making aid in cancer care, key issues that need to be tackled will be the ‘*integration of models into the EHRs and more careful evaluation of models, particularly with respect to their effects on clinical outcomes*.’ Therefore, the issue of data liberation through EHRs as a tool for making better and healthier decisions surfaces again once, this time coupled with prediction models that can synergistically work toward the same goal of better decision-making.

## Direct to consumer genetic tests drive a community-based approach to research

**Anne Wojcicki** was another of one of the big names present this year in San Francisco. Anne is the cofounder and CEO of 23&me, one of the most successful direct to consumer genetic testing companies not just in the Bay Area, but also throughout the world, and introduced the second day of the conference [11]. As Anne puts it, 23&me was founded upon two goals: first to empower individuals to be able to gain access to their own genetic information, and second to launch a new type of research that is community driven and aims at highlighting the genetic basis of drug response in order to deliver more effectively personalized medicine. While in the past Anne was often introduced as wife to Sergei Brin, one of Google cofounders, it may be the case that in a not-too distant moment Sergei will be introduced as Mr Wojcicki. 23&me has been constantly expanding since it was founded in 2007 in Mountain View, Silicon Valley, and is now not only a DTC-provider but also fostering collaborative research on the genetic causes of Parkinson’s disease (PD) and other neurodegenerative diseases. And indeed, an article recently published on PLOS Genetics and resulting from the collaboration between 23&me and the Parkinson’s Institute in Sunnyvale, CA, shows the result of a new Health 2.0 community-driven approach. The paper presented the results of a genome-wide association study (GWAS) of Parkinson’s disease counting over 3,400 cases and 29,000 controls, reported two novel genetic associations and replicated a total of 20 previously described associations [12]. To note, the study is the largest single PD GWAS cohort to date, and the first of its kind, as participation in this study took place completely online, using a collection of cases and controls derived from the customer base of the 23&me. Announced at Health 2.0 was also the **Exome 80x pilot** program, [13] where for $999 the user gets access to the raw data of his/her 50 million DNA bases at high quality (80X coverage). Over time (and more payments), the user will have access to new tools and content, as they are developed. In other words, for now the user will get the sequence but without actually knowing what to do with it, until later hopefully we will develop the science to do that. The first Exome users though can be proud to be called ‘trailblazers,’ as one can read on the website of the company. As noted by Curnutte and Testa, in a new model for genetic research where the consumer becomes a research subject, the companies (referring to the DTC companies 23&Me and Navigenics) are also constructing a mechanism for continued participation. ‘*The consumer becomes a data point, inscribed in genetic knowledge*’ [14] and also: ‘*A new social configuration is emerging, where the consumer is promised future goods through participation*’ exactly as what happens for the Exome80X pilot project.

## Web 2.0 as a positive catalyst for behavioral change

As hinted above, behavioral change is notoriously difficult: even if we know we are leading an unhealthy lifestyle and we have been told we have an increased risk for heart conditions derived from high cholesterol or hypertension, we tend to ignore the risks and not change our lifestyle. How can Web 2.0 tools help us overcome this disconnect between our intentions and actions, and facilitate behavioral change? In several ways: by providing constant feedback and tracking of ODLs and helping meet small, daily goals; and by helping the user stay ‘on the right track’ thanks to group and peer pressure, which on the internet becomes the social network and the online community. In other words, web 2.0 tools can act as a catalyst for behavioral change, i.e. a way to make it easier for people to track their behavior and to gather in groups driven by common interests and goals.

Several applications were presented at the conference that use social influence to make a positive impact on behavioral change. For example, one of the finalists of the Health 2.0 challenge *Launch!* aims to do exactly that: it is an app called ‘**Numera Social’** [15] that uses social influence for achieving behavioural change aimed at reaching better health outcomes. Another example is **Project Health Design [**16] that explores new ways to capture and integrate patient-recorded ODLs into clinical care. Among the projects particularly worth mentioning is **Chronology**, also presented at Health 2.0. [17] Nikolai ‘Kolya’ Kirienko is a student in Cognitive Science at the University of California at Berkeley and a Chron’s disease patient himself, and the Project Director. In 2000, Kirienko began keeping a comprehensive digital record of his observations of more than twelve months in the hospital, and that is when his project to ‘*translate first-hand experiences into the language of applied research*’ started, with the aim of using patient narratives to empower people with chronic diseases to make more informed health decisions in a closer collaboration with their physicians.

## Conclusions: participation across all stakeholders

**Figure 2:**
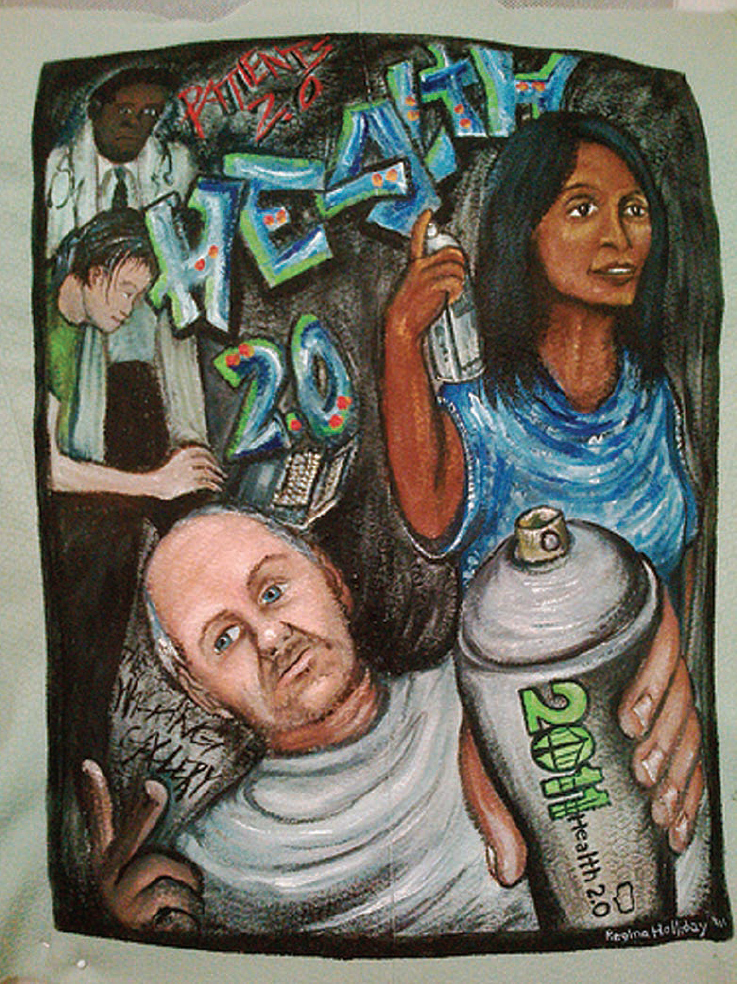
‘Health 2.0’ by Regina Holliday. Reproduced with kind permission from the artist.

The concept of participation and of involving all the stakeholders in health care was very visibly embodied by the work of Regina Holliday, who was also the designated artist of the logo for this edition of the Health 2.0 conference. In this new work, Regina is creating a mural to symbolize the interwoven interactions between doctors, patients, organizations, and medical device companies, which, if work, can lead to dramatically improved health care. (Figure 2) Regina involved all Health 2.0 attendees in this project, by asking the participants to create their own ‘tag’ or sticker to be part of the mural piece that she was painting on site. As noted by Regina, the Health 2.0 movement has much in common with the street art movement, as both sidestep traditional institutional hierarchies ‘*using a loose organizational structure supported by the tools of social media’* in their attempt to achieve their goal of a better health care. Not only do the Health 2.0 innovators act within the patient online communities, but also within the recognized medical field publications, as the example presented above of the PLOS Genetics joint publication by 23&me and the Sunnyvale Parkinson Institute shows. You can read more, and see more, of Regina’s fascinating murals on her blog, that, as elegantly captured in her words, is a place where ‘*where art, medicine, social media and pop-culture collide and create a patient voice in health information technology.*’ [18] Wish there were more voices, and places, like that out there!
